# Comprehensive analysis of forty yeast microarray datasets reveals a novel subset of genes (APha-RiB) consistently negatively associated with ribosome biogenesis

**DOI:** 10.1186/1471-2105-15-322

**Published:** 2014-09-29

**Authors:** Basel Abu-Jamous, Rui Fa, David J Roberts, Asoke K Nandi

**Affiliations:** Department of Electronic and Computer Engineering, Brunel University, Uxbridge, Middlesex, UB8 3PH UK; National Health Service Blood and Transplant, Oxford, UK; Radcliffe Department of Medicine, University of Oxford, John Radcliffe Hospital, Oxford, UK; Department of Mathematical Information Technology, University of Jyväskylä, Jyväskylä, Finland

**Keywords:** Ribosome biogenesis, Stress response, Co-expression, Co-regulation, Genome-wide analysis, Budding yeast, (Binarisation of consensus partition matrices) Bi-CoPaM

## Abstract

**Background:**

The scale and complexity of genomic data lend themselves to analysis using sophisticated mathematical techniques to yield information that can generate new hypotheses and so guide further experimental investigations. An ensemble clustering method has the ability to perform consensus clustering over the same set of genes from different microarray datasets by combining results from different clustering methods into a single consensus result.

**Results:**

In this paper we have performed comprehensive analysis of forty yeast microarray datasets. One recently described Bi-CoPaM method can analyse expressions of the same set of genes from various microarray datasets while using different clustering methods, and then combine these results into a single consensus result whose clusters’ tightness is tunable from tight, specific clusters to wide, overlapping clusters. This has been adopted in a novel way over genome-wide data from forty yeast microarray datasets to discover two clusters of genes that are consistently co-expressed over all of these datasets from different biological contexts and various experimental conditions. Most strikingly, average expression profiles of those clusters are consistently negatively correlated in all of the forty datasets while neither profile leads or lags the other.

**Conclusions:**

The first cluster is enriched with ribosomal biogenesis genes. The biological processes of most of the genes in the second cluster are either unknown or apparently unrelated although they show high connectivity in protein-protein and genetic interaction networks. Therefore, it is possible that this mostly uncharacterised cluster and the ribosomal biogenesis cluster are transcriptionally oppositely regulated by some common machinery. Moreover, we anticipate that the genes included in this previously unknown cluster participate in generic, in contrast to specific, stress response processes. These novel findings illuminate coordinated gene expression in yeast and suggest several hypotheses for future experimental functional work. Additionally, we have demonstrated the usefulness of the Bi-CoPaM-based approach, which may be helpful for the analysis of other groups of (microarray) datasets from other species and systems for the exploration of global genetic co-expression.

**Electronic supplementary material:**

The online version of this article (doi:10.1186/1471-2105-15-322) contains supplementary material, which is available to authorized users.

## Background

Advances in microarray technology have enabled measurements of expression of a vast number of genes simultaneously. Most microarray experiments consider measuring the expression values of the entire genome of a specific organism over multiple time-points, several biological developmental stages, different types of tissues, or different conditions [1]. Many different methods of microarray analysis have been designed and applied in order to address such diverse questions. Some methods aim to identify genes that are differentially expressed between certain phenotypes or conditions, which would be then predicted to participate in causing such phenotypes or in the response to such conditions [1–3]. Other methods have been proposed to look for, and model the expression of, genes that have co-ordinated expression over cell or metabolic cycles [4–7]. Moreover, various supervised and unsupervised methods have been designed to answer questions related to the co-expression of genes [8–12].

One class of supervised methods, which search for co-expressed genes, is template-based mining. Here, the microarray dataset is mined for genes whose expression profiles are similar (based on a similarity criterion, e.g. Euclidean distance) to an *a priori* known template of expression. For example, Nilsson and colleagues searched in a large number of blood-related human and mice microarray datasets for genes that are consistently co-expressed with the average expression profile of eight well-known genes that participate in haem biosynthesis [10]. Similarly, Wade and colleagues mined four budding yeast datasets for genes that are consistently co-expressed with the average expression profile of 65 previously reported ribosomal biogenesis genes [9]. Although these template based methods can confirm the consistency of co-expression of the genes matching the query template in multiple datasets, they cannot determine if there are any other clusters of genes that consistently match different templates of expression.

Amongst the classes of unsupervised methods that mine for co-expressed genes, gene clustering is the most commonly used. The objective of any of the various methods belonging to this class is to group genes into clusters such that genes included in a cluster are similar to each other while being dissimilar from the genes included in the other clusters based on a specific criterion of similarity [2]. In this way, genes are grouped into subsets of co-expressed genes. Examples of methods used for gene clustering are k-means [11], hierarchical clustering (HC) [8], self-organizing maps (SOMs) [13, 14] and self-organizing oscillator networks (SOON) [15], as well as ensemble methods, e.g. relabeling and voting [16], co-association matrix [17], hypergraph methods [18], and the recently proposed binarisation of consensus partition matrices (Bi-CoPaM) [19–21].

A major drawback of most clustering methods is that they impose the constraint that each gene must be exclusively assigned to one and only one cluster. Thus, feeding genome wide data to such clustering methods always produces clusters that include all of the genes in this genome; therefore, the size and complexity of the data are not decreased significantly. We have tackled this problem by our recently published unconventional ensemble clustering method (Bi-CoPaM), which provides a platform that allows for generating conventional complementary clusters in which each gene is exclusively assigned to a single cluster, as well as unconventional clusters such as wide overlapping clusters in which genes can be simultaneously assigned to multiple clusters, and tight clusters which leave many genes unassigned to any cluster [19, 20]. Producing such varying forms of unconventional clusters allows tuning, such that different gene discovery studies can tune the Bi-CoPaM to produce the particular form of clusters that helps in answering that study’s specific questions. Moreover, the tuneable partitions produced by the Bi-CoPaM are based on the consistency of co-expression of a set of genes across multiple microarray datasets and when clustered by various clustering methods [19, 20]. The Bi-CoPaM method does not combine the profiles of the genes in multiple datasets in order to analyse them collectively. It rather achieves this collective analysis by examining each dataset independently and then combining their results into a single consensus result [19, 20].

Wade and colleagues identified a subset of genes consistently co-expressed with a template of 65 ribosomal biogenesis genes in four different datasets [9]. That subset was found to be enriched with rRNA processing and ribosomal biogenesis genes (RRB), and was found to be up-regulated when released from cell-cycle arrest while being down-regulated under stress [9]. Other studies have identified RRB-enriched subsets of genes with profiles that are consistently positively correlated with growth and negatively correlated with stress [22, 23]. On the other hand, other subsets of genes, mainly enriched with stress response genes, were identified as negatively correlated with growth and positively correlated with stress [22, 23]. The regulation of such subsets of datasets has been discussed by various studies which listed different confirmed or potential regulators such as Tod6p, Stb3p, and Sfp1p for RRB genes, and Msn2/4p, Rgt1p, and Adr1p for stress response genes [9, 22–25]. Also, the relations between growth rate and stress resistance, as well as between the expression of RRB genes and other regulons such as ribosomal proteins and cell cycle genes were discussed while considering signal transduction pathways (e.g. TOR1 and Ras/PKA pathways) or transcription factors as regulatory connections [9, 23, 24]. Each of those studies considered one or few datasets to obtain its conclusions.

In this study, we adopt a novel approach of the Bi-CoPaM method to analyse genome-wide data from forty microarray yeast datasets from a wide range of biological conditions and contexts in order to identify the subsets of genes that are consistently co-expressed in *Saccharomyces cerevisiae* budding yeast under such various conditions [19, 20]. We investigate if the RRB genes are consistently co-expressed in a wider range of conditions than those investigated by previous studies [9, 22, 23]. Moreover, we explore if there are other novel subsets of budding yeast genes that are consistently co-expressed over such wide range of different microarray datasets and, if so, we investigate their previous characterisations and known function(s), and we draw hypotheses regarding their regulation as well as the potential roles of their poorly understood genes in cell biology.

## Methods

### Bi-CoPaM

**Figure 1:**
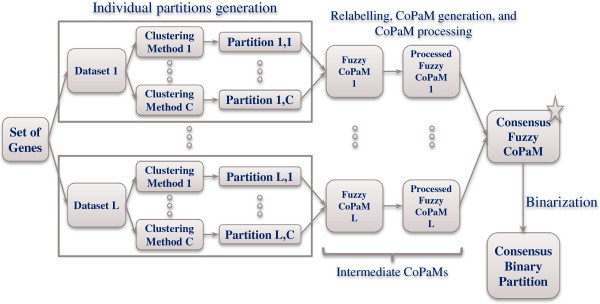
**The pipeline of steps in the Bi-CoPaM method.**

The Bi-CoPaM method consists of four main steps (Figure 1) [19, 20]:

1. Generation of many partitions for the same set of genes by applying various clustering methods over the expression profiles of these genes from multiple microarray datasets.
2. Relabelling the generated partitions such that each cluster from one partition is matched with its corresponding cluster from every other partition.
3. Generation of the fuzzy consensus partition matrix (CoPaM) by element-by-element averaging of the relabelled partitions.
4. Binarization of the CoPaM by one or more of the six tunable binarization techniques proposed in [19].
  To amplify the variation in cluster assignment caused by the differences in microarray datasets over the one caused by the differences amongst clustering methods, the partitions generated by applying different clustering methods over any single microarray dataset are first combined into a single intermediate fuzzy consensus partition matrix (CoPaM) whose membership values are processed by pushing them towards the binary values of zero and one (Figure  1); this is mathematically formulated as

where *u*_*i,j*_ and  are the fuzzy membership values for the *j*^*th*^ gene in the *i*^*th*^ cluster before and after processing, respectively, and *m*_*j*_ is mean of the fuzzy membership values of the *j*^*th*^ gene in all of the clusters in which it has non-zero values. After all CoPaM matrices from all of the microarray datasets are generated and processed as described herein, they are combined to produce the final CoPaM, which is then binarised to produce the final binary partitions.

The six binarization techniques scrutinize the CoPaM in different ways to produce binary partitions with different features. Our concentration in this study is on the *difference threshold binarization (DTB)* technique and its two extreme special cases *maximum value binarization (MVB)* and *intersection binarization (IB)*.

The MVB technique assigns each gene to the cluster in which it has its maximum fuzzy membership; this generates conventional complementary clusters in which each gene is exclusively assigned to one and only one cluster. The DTB technique imposes a stricter policy; it assigns this gene to that maximum-membership cluster only if the closest cluster competing on this gene has a fuzzy membership value which is lower than the maximum by at least the value of the parameter (δ). Otherwise, the gene is not considered clearly belonging to a specific cluster and is unassigned from all of the clusters accordingly. When this DTB parameter (δ) is zero, it is equivalent to the MVB technique. Tighter clusters with more unassigned genes are obtained when δ is increased until it reaches one. When its value is one, only genes that have been consensually assigned to the same clusters by all of the single partitions are preserved; all of the other genes are left unassigned. This tightest case is equivalent to the IB technique.

### Mean Squared Error (MSE) metric

The mean squared error (MSE) metric has been used by many studies to evaluate the quality of the generated clusters so that comparisons between different methods can be performed [26, 27]. We adopt the MSE metric for evaluating the generated clusters.

Because the total number of genes assigned to the clusters by Bi-CoPaM at any specific tightness level is variable, we use a normalized MSE measure to be *per gene*. The *MSE*_*cluster*_ metric which quantifies the total MSE for the *k*^*th*^ cluster is defined as:


Where *N* is the number of dimensions (time-points) in the dataset, *M*_*k*_ is the number of genes in the *k*^*th*^ cluster, *C*_*k*_ is the set of zero-centred unity-standard-deviation genetic expression profiles {*x*_*i*_} for the genes in the *k*^*th*^ cluster, and *z*_*k*_ is the mean expression profile for the genes in the *k*^*th*^ cluster.

If multiple datasets were used for clustering, genes profiles and the clusters centroids will vary from one dataset to another for the same partition. In this case, the MSE metric can be calculated multiple times for each dataset and then averaged over them.

### Datasets & experimental procedures

In this study, we consider forty recent *Saccharomyces cerevisiae* microarray datasets which were generated by using the Affymetrix yeast genome 2.0 array in the last six years, and include at least four different conditions or time-points. Although choosing datasets generated by using the same array is not a condition for Bi-CoPaM analysis, it allows for more genes to be included in the analysis as some genes might not be represented by probes in all types of arrays, and therefore have to be discarded from the analysis in such a case. Each of these datasets measures the genetic expression of the entire yeast genome (5,667 genes) over multiple time-points or conditions. The details of the datasets are listed in Table 1. The datasets span a wide range of biological conditions such as cell-cycle, stress response, mutated strains growth, treatment with various types of agents, and others. The 5,667 genes are listed in Additional file 1: Table S1.

**Table 1:** **Budding yeast microarray datasets**

ID	GEO accession	Year	N	Description	Ref.
D01	GSE8799	2008	15	Two mitotic cell-cycles (w/t).	[28]
D02	GSE8799	2008	15	Two mitotic cell-cycles (mutated cyclins).	[28]
D03	E-MTAB-643*	2011	15	Response to an impulse of glucose.	[14]
D04	E-MTAB-643*	2011	15	Response to an impulse of ammonium.	[14]
D05	GSE54951	2014	6	Response of *dal80Δ* mutant yeast to oxidative stress induced by linoleic acid hydroperoxide.	-
D06	GSE25002	2014	9	Osmotic stress response and treatment of transformants expressing the *C. albicans* Nik1 gene.	-
D07	GSE36298	2013	6	Mutations of OPI1, INO2, and INO4 under carbon-limited growth conditions.	[29]
D08	GSE50728	2013	8	120-hour time-course during fermentation.	-
D09	GSE36599	2013	5	Stress adaptation and recovery.	[30]
D10	GSE47712	2013	6	Combinations of the yeast mediator complex’s tail subunits mutations.	[31]
D11	GSE21870	2013	4	Combinations of mutations in DNUP60 and DADA2.	-
D12	GSE38848	2013	6	Various strains under aerobic or anaerobic growth.	[32]
D13	GSE36954	2012	6	Response to mycotoxic type B trichothecenes.	[33]
D14	GSE33276	2012	6	Response to heat stress for three different strains.	-
D15	GSE40399	2012	7	Response to various perturbations (heat, myriocin treatment, and lipid supplement).	-
D16	GSE31176	2012	6	W/t, *rlm1Δ*, and *swi3Δ* cells with or without Congo Red exposure.	[34]
D17	GSE26923	2012	5	Varying levels of GCN5 F221A mutant expression.	[35]
D18	GSE30054	2012	31	CEN.PK122 oscillating for two hours.	-
D19	GSE30051	2012	32	CEN.PL113-7D oscillating for two hours.	[36]
D20	GSE30052	2012	49	CEN.PL113-7D oscillating for four hours.	[36]
D21	GSE32974	2012	15	About 5 hours of cell-cycle (w/t).	[37]
D22	GSE32974	2012	15	About 4 hours of cell-cycle (mutant lacking Cdk1 activity).	[37]
D23	GSE24888	2011	5	Untreated yeast versus yeasts treated with *E. arvense* herbs from the USE, China, Europe, or India.	-
D24	GSE19302	2011	6	Response to degron induction for w/t and nab2-td mutant.	[38]
D25	GSE33427	2011	5	Untreated w/t, and wt/t, *yap1Δ*, *yap8Δ*, and double mutant treated with AsV.	[39]
D26	GSE17716	2011	7	Effect of overexpression and deletion of MSS11 and FLO8.	[40]
D27	GSE31366	2011	4	Presence and absence of mutli-inhibitors for parental and tolerant strains.	-
D28	GSE26171	2011	4	Response to patulin and/or ascorbic acid.	[41]
D29	GSE22270	2011	4	PY1 and Met30 strains in room temperature or 35 C.	-
D30	GSE29273	2011	4	Time-series during yeast second fermentation.	-
D31	GSE29353	2011	5	Different haploid strains growing in low glucose medium.	[42]
D32	GSE21571	2011	8	Different combinations of mutations in HTZ1, SWR1, SWC2, and SWC5.	[43]
D33	GSE17364	2010	4	Untreated w/t and Slt2-deficient yeasts, or treated with sodium arsenate for two hours.	[44]
D34	GSE15352	2010	8	24-hour time-course of yeast grown under a low temperature (10 C).	[45]
D35	GSE15352	2010	8	24-hour time-course of yeast grown under a normal temperature (28 C).	[45]
D36	GSE15352	2010	8	24-hour time-course of yeast grown under a high temperature (37 C).	[45]
D37	GSE16799	2009	21	UC-V irradiation of w/t, *mig3Δ*, *SNF1Δ*, *RAD23Δ*, *RAD4Δ*, and *snf1Δrad23Δ*.	[46]
D38	GSE16346	2009	4	BY474 cells grown to mid-log under presence versus absence of L-carnitine and/or H_2_O_2_.	-
D39	GSE14227	2009	10	Two hours of wild-type yeast growth.	[47]
D40	GSE14227	2009	9	Two hours of *sch9Δ* mutant yeast growth.	[47]

The first column shows the unique identifier which is used hereinafter to refer to each of these datasets. The second to the sixth columns respectively show the Gene Expression Omnibus (GEO) accession number, the year in which the dataset was published, number of time-points or conditions after replicate summarisation, dataset description, and reference.

*D03 and D04 have accession numbers in the European Bioinformatics Institute (EBI) repository rather than GEO accession numbers.

These 5,667 genes were clustered into sixteen clusters by k-means with Kauffman initialisation (KA) [48], self-organising maps (SOMs) with bubble neighbourhood and four-by-four grid [13], and hierarchical clustering (HC) with Ward’s linkage [8]. This was applied to their profiles from all of the forty datasets. The generated partitions were combined into a single consensus partition matrix (CoPaM) as explained in Bi-CoPam where a min-min approach was adopted for relabeling at the CoPaM generation step. The final CoPaM was binarised by the difference threshold binarization (DTB) technique with δ values ranging from zero to one and then analysed by the MSE metric described in Mean Squared Error (MSE) metric. Prior to clustering, the datasets were normalized by quantile normalization [49]. Then each gene’s expression profile was shifted and scaled to be zero-mean and unity standard deviation. Also, when many replicates exist for the same time-point or condition, they are summarised by considering their median value.

## Results

The numbers of genes in the sixteen clusters at all of the varying δ values are shown in Table 2. Clusters were ordered based on their tightness such that those clusters that preserve at least seven genes up to higher values of δ are considered tighter. When many clusters preserve at least seven genes up to the same value of δ, they are ordered based on the number of genes they include at that level. The number ‘seven’ is just used for ordering and is not a critical parameter; if it had been set to ‘ten’ instead for example, no significant change in cluster ordering would have be observed. The complete lists of genes included in each of these clusters at all of the δ values are provided in Additional file 1: Table S1.

**Table 2:** **Numbers of genes included in each of the 16 clusters at all of the considered δ values**

Tightness	δ	Cluster															
		C1	C2	C3	C4	C5	C6	C7	C8	C9	C10	C11	C12	C13	C14	C15	C16
Complementary	0.0	1085	1457	610	655	592	268	303	175	175	154	143	92	51	49	29	10
	0.1	516	394	84	105	79	12	9	3	1	2	2	0	0	0	0	0
	0.2	344	47	17	14	2	0	0	0	0	0	0	0	0	0	0	0
	0.3	257	0	0	2	0	0	0	0	0	0	0	0	0	0	0	0
	0.4	164	0	0	0	0	0	0	0	0	0	0	0	0	0	0	0
	0.5	79	0	0	0	0	0	0	0	0	0	0	0	0	0	0	0
	0.6	22	0	0	0	0	0	0	0	0	0	0	0	0	0	0	0
	0.7	0	0	0	0	0	0	0	0	0	0	0	0	0	0	0	0
	0.8	0	0	0	0	0	0	0	0	0	0	0	0	0	0	0	0
	0.9	0	0	0	0	0	0	0	0	0	0	0	0	0	0	0	0
Tightest	1.0	0	0	0	0	0	0	0	0	0	0	0	0	0	0	0	0

### MSE analysis

**Figure 2:**
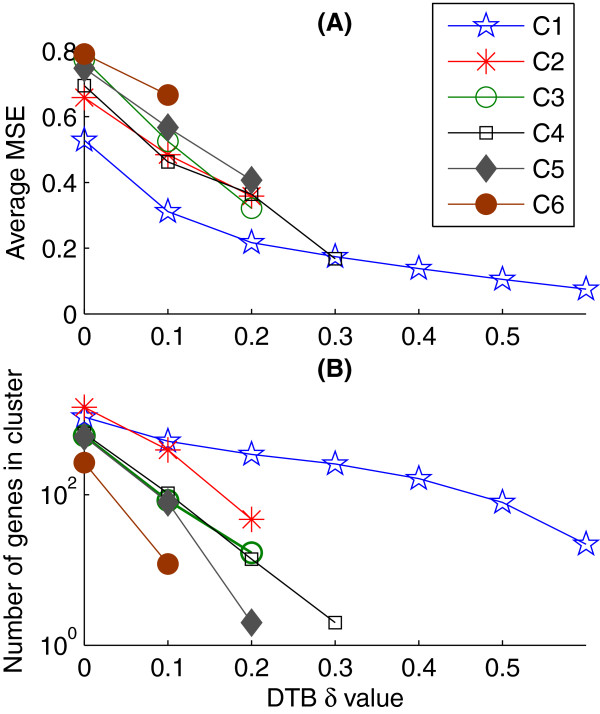
**Average MSE values and the number of genes included in the tightest six clusters at all of the adopted δ values. (A)** Average MSE values and **(B)** number of genes included.

The MSE values for each of the tightest six clusters were calculated at all of the DTB δ values as explained in Mean Squared Error (MSE) metric. Each of these values was calculated based on the forty datasets and then averaged and plotted in Figure 2(A). Figure 2(B) shows the numbers of genes included in each of these six clusters at all of the δ values. Missing points in both plots represent empty clusters.

We have considered the mean standard error (MSE) evaluation metric in tandem with the number of genes included in the clusters to choose a few clusters for further analysis and discard the other ones. The objective here is to minimise the MSE values while maximising the number of genes included in the clusters. This approach overcomes the dependency of MSE values on the numbers of genes included in the clusters. As can be seen in Figure 2(A) and (B), the cluster C1 shows significantly lower (better) values of MSE while including significantly higher numbers of genes. The cluster C2 comes next to C1 in terms of having lower MSE values with more genes.

On the other hand, while the clusters C3 and C4 have comparative MSE values at δ = 0.2 with C2 (Figure 2 (A)), they have significantly lower numbers of genes (17 and 14 genes respectively for C3 and C4 in comparison with 47 in C2; see Table 2). Furthermore, the clusters C5 and C6 are significantly worse (higher MSE values with fewer genes) than the first four clusters (Figure 2). While the average MSE values for the seventh to the sixteenth clusters have not been included in that Figure, the numbers of genes included in these clusters at relatively lower levels of tightness, as shown in Table 2, are sufficient to filter them out. Therefore, we have considered the clusters C1 and C2 for further analysis in this study.

### Average expression profiles

**Figure 3:**
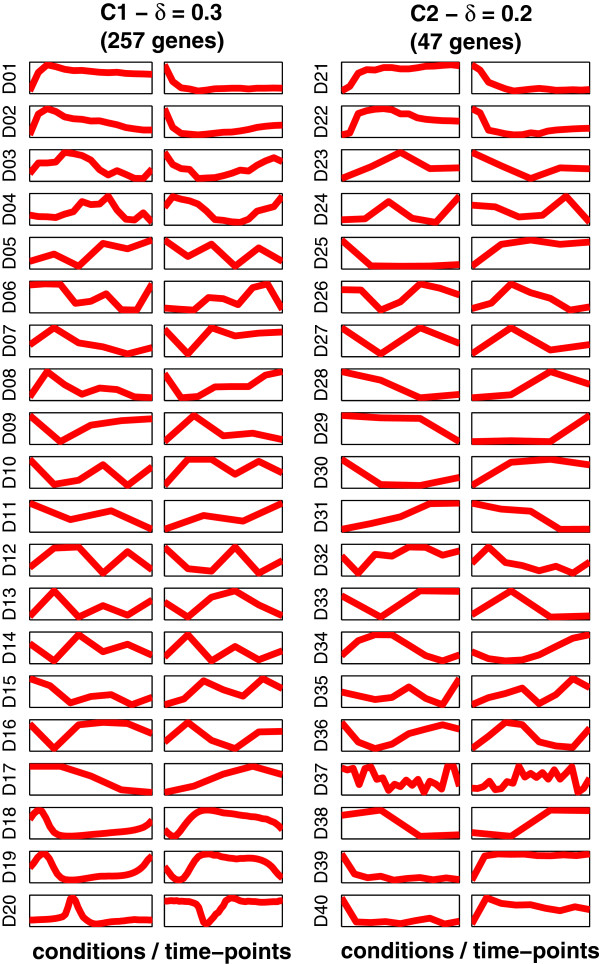
**Average expression profiles for the clusters C1 and C2 at DTB with the respective δ values of 0.3 and 0.2, based on all of the forty datasets.** Each column of plots represents a cluster and each row represents a dataset.

The average expression profiles for the clusters C1 and C2 at DTB with δ = 0.3 and 0.2 respectively, in each of the forty datasets are plotted in Figure 3. For clarity, error bars have been suppressed as the information, which they provide can be obtained from the MSE analysis in Figure 2 and the plots in Additional file 2: Figure S1, which shows the expression profiles of all of the genes in these two clusters at various δ values.

Detailed scrutiny of Figure 3 leads to the general observations that the first cluster, C1, is up-regulated when cells are released from stress conditions such as nutrient limitation; they are down-regulated when stress conditions are re-imposed. Most interestingly, the cluster C2 shows opposite average expression profiles in almost all of the forty datasets to the average profiles of cluster C1 with no phase shift, *i.e.* with neither profile leading or lagging the other; its genes are up-regulated under stress conditions and down-regulated under growth conditions. It is interesting, but had not been anticipated at the time of experimental design before obtaining the results, that the two most consistently co-expressed clusters of genes in budding yeast show such clear opposite expression profiles across large number of datasets.

To assess that observed opposite co-expression quantitatively, we have calculated the Pearson’s correlation values between the average expression profiles of C1 at δ = 0.3 and C2 at δ = 0.3 from each of the forty datasets. A very strong negative correlation has been found, that is lower than the value of -0.75 at 37 out of 40 datasets and never exceeds the value of -0.6 except at a single outlier dataset, D35. This strong negative correlation is consistent even when the δ values are varied. For instance, when considering C1 at the δ values of 0.2 and 0.4, the calculated correlation values are lower than -0.75 at 38 and 36 out of 40 datasets, respectively. Even when considering C2 at δ = 0.1, the case at which its size is many folds larger than at δ = 0.2 (394 genes versus 84), 35 out of 40 datasets show strong negative correlation with values lower than -0.75, and only couple of datasets exceed the value of -0.7. The single outlier dataset D35 has consistently shown notably weaker negative correlation at all of the aforementioned δ values. These experiments demonstrate the robustness of our observation that C1 and C2 are consistently negatively correlated.

### Promoters enrichment analysis

**Figure 4:**
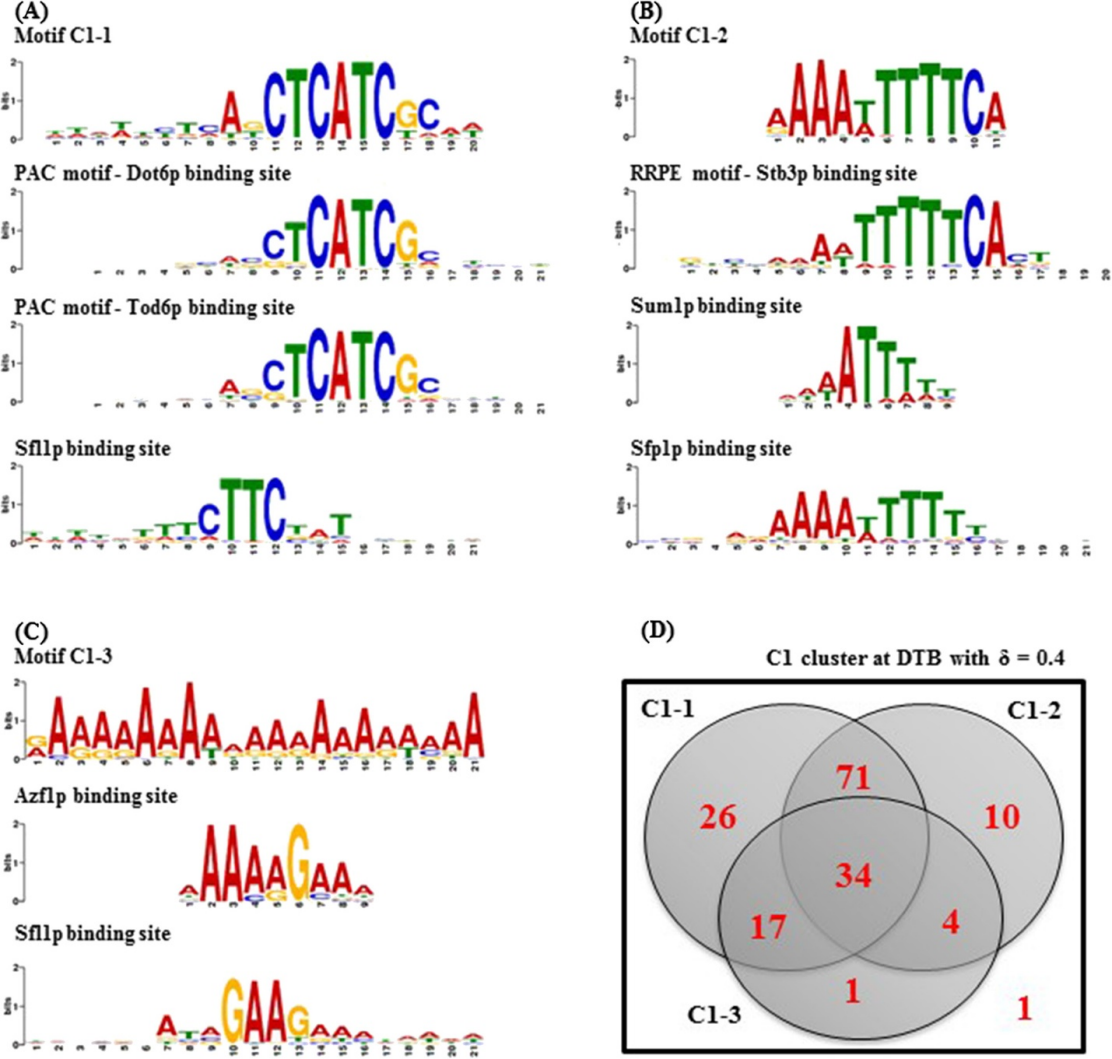
**Upstream sequence analysis for the cluster C1. (A)**, **(B)**, and **(C)** show the motifs C1-1, C1-2, and C1-3 respectively and their highly matched known transcription factors’ binding sites. **(D)** is a Venn diagram that shows the numbers of genes’ upstream sequences in C1 that contain each of these three motifs.

Because co-expression over large number of different microarray datasets strongly indicates co-regulation, we have analysed the upstream DNA sequences for the genes in the clusters C1 and C2 to explore potential common transcription factors’ binding sites. We have used the MEME tool [50, 51] to search for the most enriched DNA sequence motifs within the 300 upstream base-pairs of the 164 genes included in C1 at DTB with δ = 0.4. The three discovered motifs, which we label as C1-1, C1-2, and C1-3 respectively, were then fed to the TOMTOM tool [52, 53] to mine for previously known motifs with high similarity. The first motif, with an E-value of 3.3 × 10^-333^, was found to be the PAC motif, which is the binding site of the two paralogous transcription factors Dot6p and Tod6p with p-values of 2.1 × 10^-5^ and 1.4 × 10^-4^, respectively, and it significantly matches the binding site of the transcription factor Sfl1p with a p-value of 1.3 × 10^-4^ (Figure 4(A)). The E-value estimates the expected number of motifs with the given probability or higher, and with the same width and site count, that would be found in a set of random sequences of a similar size. The second motif, with an E-value of 2.2 × 10^-115^, was found to be the RRPE motif, which is the binding site of the transcription factor Stb3p with a p-value of 8.9 × 10^-7^ (Figure 4(B)); it also significantly matches the binding sites of the transcription factors Sum1p and Sfp1p with p-values of 2.7 × 10^-5^ and 3.2 × 10^-5^, respectively (Figure 4 (B)). The third motif, with an E-value of 3.2 × 10^-63^, was found to match the binding sites of the transcription factors Azf1 and Sfl1p with p-values of 1.3 × 10^-4^ and 2.0 × 10^-4^, respectively (Figure 4(C)). The three motifs were respectively found in the upstream sequences of 148, 119, and 56 genes out of 164 possible ones. Figure 4 (D) is a Venn diagram, which shows the numbers of genes the upstream DNA sequences of which contain each of these three motifs.

**Figure 5:**
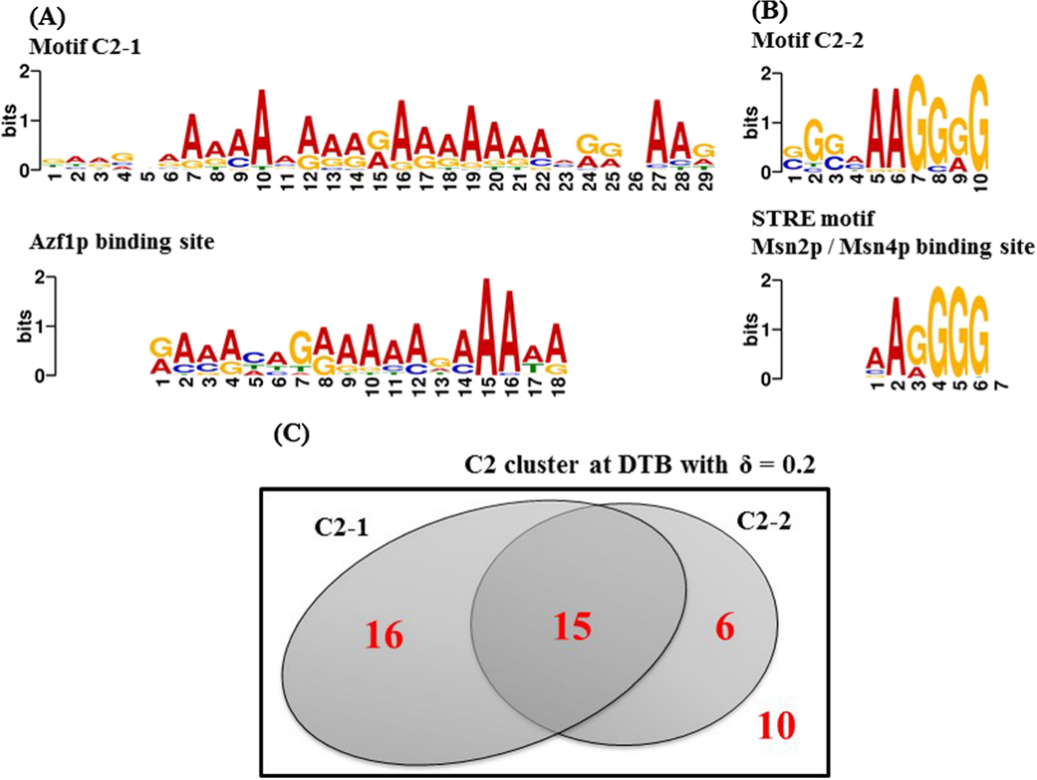
**Upstream sequence analysis for the cluster C2. (A)** and **(B)** show the motifs C2-1 and C2-2 respectively and their highly matched known transcription factors’ binding sites. **(C)** is a Venn diagram that shows the numbers of genes’ upstream sequences in C2 that contain each of these two motifs.

Similarly, the MEME tool was used over the 47 genes included in the cluster C2 at DTB with δ = 0.2. The logos of the two discovered motifs, which we label as C2-1 and C2-2, are shown in Figure 5 (A) and (B), respectively. The E-values for the two motifs are 1.6 × 10^-23^ and 5.3 × 10^-4^ respectively, and they were found in the upstream sequences of 31 genes and 21 genes, out of 47 genes in C2 at DTB with δ = 0.2 (Figure 5(C)). A third motif was found by the MEME tool in this cluster but with the high E-value of 2.8 × 10^+1^ and in the upstream sequences of 13 genes only; therefore it has been discarded from further analysis. The motifs C2-1 and C2-2 were then fed to the TOMTOM tool [52, 53] to mine for previously known motifs that have high similarity to them. The motif C2-1 was found to match the binding site of the transcription factor Azf1p (p-value 5.4 × 10^-6^), while C2-2 was found to match the STRE element which is the binding site of the transcription factor Msn4p (p-value 5.4 × 10^-4^) and its paralogue Msn2p (p-value 6.2 × 10^-4^). The logos of the binding sites of these transcription factors aligned with the discovered motifs are shown in Figure 5(A) and (B), respectively.

### GO analysis

To link our observations over the clusters’ expression profiles with biological terms, we have performed Gene Ontology (GO) analysis [54] over the clusters C1 and C2 at different tightness levels by using the GO Term Finder tool [55], and the GO Slim Mapper tool [56]. The most enriched GO process terms in these clusters, as well as the numbers of genes annotated with the GO term “biological process unknown”, are listed in Table 3. Additional file 3: Table S2 and Additional file 4: Table S3 include the complete GO Term Finder and GO Slim Mapper tools results, respectively, for the clusters C1 and C2 at all of the values of δ at which they are not empty.

**Table 3:** **Most enriched GO terms in the clusters C1 and C2 at various levels of tightness**

	GO process	Back. frequency	δ = 0.1		δ = 0.2		δ = 0.3		δ = 0.4		δ = 0.5	
			Freq.	P-val.	Freq.	P-val.	Freq.	P-val.	Freq.	P-val.	Freq.	P-val.
C1	Ribosome biogenesis	411/7167	210/516	E-140	183/344	E-146	153/257	E-129	124/164	E-123	65/79	E-66
	Biological process unknown*	1189/6334	46/516		26/344		17/257		9/164		4/79	
C2	Response to oxidative stress	101/7167	23/394	E-6	6/47	E-3						
	Oxidation-reduction process	174/7167	33/394	E-7	3/47	>E-1						
	Biological process unknown*	1189/6334	114/394		12/47							

*The enrichment of the “biological process unknown” term has been found by the GO Slim Mapper tool rather than the GO Term Finder tool. Note that the p-value is only provided by the GO Term Finder tool.

The cluster C1 is extraordinarily highly enriched with genes that participate in ribosome biogenesis and rRNA processing (RRB), and it includes a small number of genes of unknown biological process. In contrast, the genes included in the cluster C2 include a large group of unknowns (12 genes, 25.5%, with unknown biological process out of 47 in C2 at δ = 0.2, and 114 out of 394, 28.9% at δ = 0.1), and even the genes with currently known processes do not show dominant enrichment for any single process. Relatively, the most enriched known biological processes within the 47 genes included in this cluster at δ = 0.2 are response to oxidative stress (six genes, 12.8%) and oxidation-reduction (three genes, 6.4%); no genes are shared between these two processes. Other processes with which some genes in this cluster have been associated are lipid metabolic process (four genes, 8.5%), carbohydrate metabolic process (four genes, two of which has also been associated with oxidation-reduction, and one with response to oxidative stress), cellular amino acid metabolic process (four genes, one of which has also been associated with response to oxidative stress), protein phosphorylation (three genes, one of which has also been associated with oxidation-reduction), mitochondrial organisation (two genes), cofactor metabolic process (two genes), regulation of cell cycle (two genes, one of which has also been associated with oxidation-reduction), endocytosis (two genes, one of which has also been associated with protein phosphorylation), and response to heat (two genes, one of which has also been associated with protein phosphorylation).

**Figure 6:**
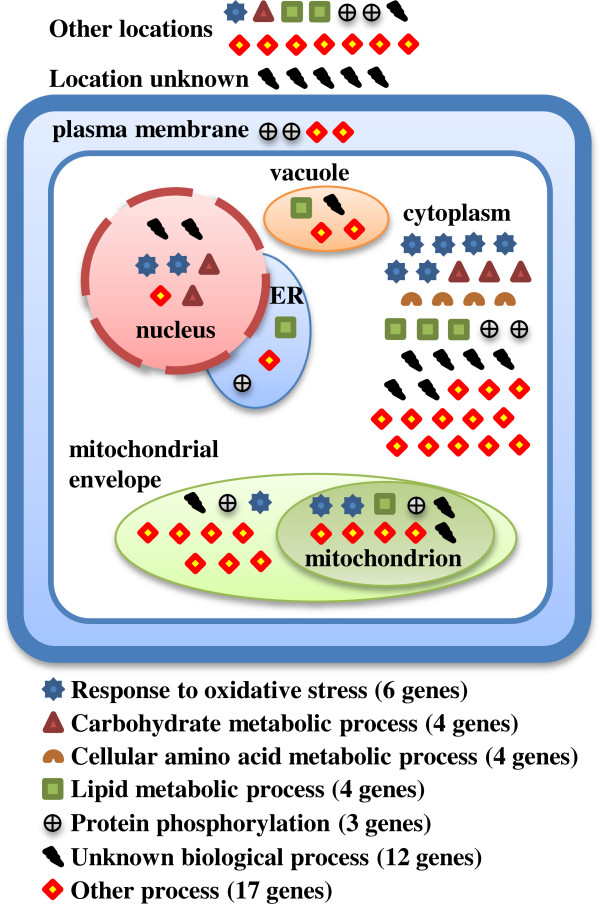
**The distribution of the 47 genes included in C2 at DTB with δ = 0.2 based on the biological processes with which they have been associated and over the major cellular components.** Note that any single gene might be found in multiple cellular components, and thus the total number of gene markers in the Figure does not directly correspond to the total number of genes considered.

We have also searched for the enrichment of the cellular components in which the C2 genes included at DTB with δ = 0.2 localise. The complete lists of results are provided in Additional file 5: Table S4. Figure 6 shows the distribution of the genes included in C2 at that tightness level over main cellular components while marked based on their biological processes. It can be seen that there is a large distribution of processes as well as components with no single process or component dominating.

In conclusion, we name the subset of genes in C2 as “anti-phase with ribosome biogenesis regulon”, or *APha-RiB regulon*. This is because its main characterising feature is its consistently opposite expression with the RRB regulon (C1).

### Gene network analysis

**Figure 7:**
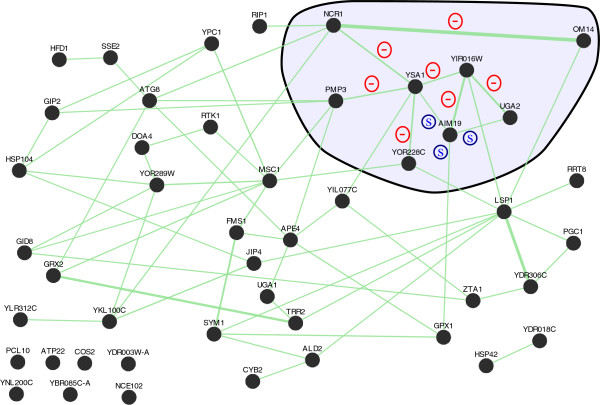
**Genetic interaction network between the genes in the APha-RiB regulon (C2 at DTB with δ = 0.2).** A sub-network of eight genes is highlighted and the types of genetic interactions between its genes are labelled. This is the same sub-network which is highlighted in Figure 8. A genetic interaction exists between two genes if the impact of perturbing both genes is different from the additive impact of perturbing each gene individually. A positive genetic interaction is that in which perturbing both genes results in a higher fitness, i.e. a weaker defect, than the additive defect of perturbing each one individually. On the other hand, a negative genetic interaction exists when the defect caused by perturbing both genes is stronger than the additive defect caused by perturbing each gene individually. A similar profile (S) genetic interaction indicates high correlation between both genes’ genetic interaction profiles with the rest of the genes.

**Figure 8:**
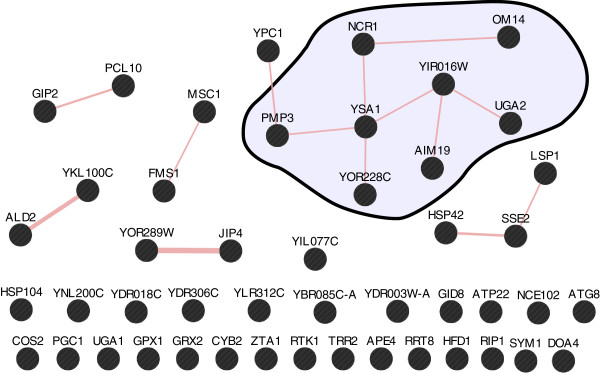
**Protein-protein physical interaction network between the products of the genes in the APha-RiB regulon (C2 at DTB with δ = 0.2).** Each node represents a gene, and a link between any two nodes represents the existence of a physical interaction between the products of those genes, i.e. between the proteins which are encoded by those genes. A relatively highly connected sub-network of eight genes is highlighted for more discussion in the main text; this is the same sub-network highlighted in Figure 7.

GeneMANIA is a tool which mines a database of various types of interactions identified by high-throughput studies in the literature to draw networks of interactions for a subset of query genes [57]. By using this tool, we have obtained networks of genetic interactions (Figure 7) and protein-protein physical interactions (Figure 8) between the 47 genes included in the APha-RiB regulon (cluster C2 at δ = 0.2).

We have also used GeneMANIA to find the network of genetic co-expression between the 47 APha-RiB genes in order to validate their consistent co-expression. The produced network contains 962 co-expression links out of 1,081 possible ones (89%) in this undirected graph of 47 nodes. To test the statistical significance of these figures, we randomly generated ten different groups of genes, each of which has 47 genes, and fed them to the GeneMANIA tool. The average number of co-expression links was 380 links with a standard deviation of 32. Therefore, by assuming a normal distribution, the p-value of having 962 links between 47 nodes is 6.7 × 10^-73^, which proves the validity of including those 47 genes in a single cluster.

A sub-network of eight genes is highlighted in Figure 7 and Figure 8 because they have high connectivity in both genetic and protein-protein physical interactions networks. The types of the genetic interactions between those eight genes are also labelled in Figure 7. Based on the high-throughput study by Costanzo and colleagues [58], two genes have positive genetic interaction between them if the effect of perturbing both genes is higher than the additive effect of perturbing each gene individually. Similarly, they have negative genetic interaction if the effect of perturbing both of them is less than the additive effect of perturbing each one of them individually. If the effect of perturbing both of them is similar to the additive effect of perturbing each of them individually, they do not have genetic interaction. The interactions labelled with (S) in Figure 7 indicate that there is high correlation between the genetic interaction profiles of those two genes with the other genes in the yeast genome.

It is interesting that, within the selected sub-network, there is a perfect one-to-one correspondence between protein-protein physical interactions and negative genetic interactions (Figure 7 and Figure 8). When this is added to their consistent co-expression over forty different and recent datasets, it can be hypothesised that they are related functionally, which can be tested in future biological studies.

### Experiments with different numbers of clusters

We have repeated the Bi-CoPaM experiment over the same datasets but with different K values other than sixteen, *i.e.* different numbers of clusters. We tried the K values 8, 9, 10, 18, 24, 30, and 40. At all of the given K values, the cluster RRB was found as the absolutely tightest cluster with very high similarity in its gene content to the cluster found at K = 16. At the K values of 8, 9, and 10, the results have shown that the second tightest cluster is similar to the APha-RiB regulon found in this study, while at the K values of 18 and 24, it was split into two smaller clusters. Moreover, at the K values of 30 and 40, many other small tight clusters appeared but many of them are redundant in terms of their expression profiles and should be rather combined. Interestingly, no other significant cluster found in any of those results. This experiment shows that our proposed approach of applying the Bi-CoPaM method to genome-wide datasets is robust over a wide range of K values.

## Discussion

Our results, based on a Bi-CoPaM-analysis of forty different and recent yeast microarray datasets each measuring the genetic expression of the yeast genome (~6000 genes) over multiple time-points or conditions, illustrate that the two most consistently co-expressed subsets of *S. cerevisiae* genes are the ribosomal biogenesis regulon (RRB) and a subset of genes which is in anti-phase (negative correlation) with ribosome biogenesis (APha-RiB). The genes in the latter subset have thus far been considered apparently unrelated as it includes a large proportion of genes of unknown function. We propose that expression of APha-RiB subset of genes is associated with control of cellular processes required under general stress conditions. These findings strongly suggest that a common machinery exists to regulate both subsets at their transcriptional level and we propose candidate regulators of these subsets of genes. Finally, our results demonstrate a successful novel application of the Bi-CoPaM method to analyse gene expression over multiple genome-wide datasets, which could be generalised to other groups of microarray datasets from budding yeast and indeed other species.

### Ribosome biogenesis genes are the most consistently co-expressed in budding yeast

The fact that ribosome biogenesis genes are highly consistently co-expressed across various conditions has been reported previously by different studies which adopted different approaches [9, 22, 23, 59]. Wade and colleagues mined four microarray datasets for the subset of genes consistently co-expressed with a template of genes known to participate in rRNA and ribosome biogenesis (RRB) [9]. Their results unveiled a set of 188 genes, which were consistently co-expressed with the RRB query genes, and their upstream sequences were enriched (158/188) with the PAC and/or RRPE motifs [9]. Brauer and colleagues produced an expression dataset which includes six sub-series of yeast that experiences different levels of growth under different types and levels of stress [22]. They then identified two subsets of genes which are consistently, positively or negatively, linearly correlated with growth rate [22]. Roy and colleagues also identified modules (subsets) of genes which are positively or negatively correlated with heat stress in a conserved manner across eight species of *Ascomycota* yeast; indeed one of them is *Saccharomyces cerevisiae* [23]. The cluster C1 is highly similar to the subsets of genes positively correlated with growth and highly enriched with ribosome biogenesis in those three studies, but in most cases C1 has higher enrichment and/or lower false-positive rate discovery (Additional file 6: Figure S2). Therefore, our results recapture this biological fact while defining a more focused subset of genes based on forty different datasets.

Many other previous studies have also observed co-regulation of the ribosome biogenesis genes in responses to environmental conditions such as being up-regulated when cells are released from stress conditions such as alpha factor arrest and nutrient limitation [9, 14], or down-regulated when stress conditions are re-imposed [6, 60], or cyclically regulated during the yeast metabolic cycle (YMC) [6].

### A novel subset of largely unknown genes (APha-RiB) is consistently in anti-phase (Oppositely Co-Regulated) with ribosome biogenesis genes

One of the most striking findings of our *in silico* experiments is the discovery of the C2 cluster of genes, which are consistently oppositely co-regulated with RRB genes over forty different and recent datasets with no phase shift (*i.e.* their average expression profile neither lags nor leads the average time profile of the RRB genes), as can be seen in Figure 3. Therefore, we have labelled this subset of genes, which is in anti-phase with ribosome biogenesis, as the *APha-RiB regulon*. This suggests that the APha-RiB and the RRB regulons may be transcriptionally oppositely regulated by some common machinery.

**Figure 9:**
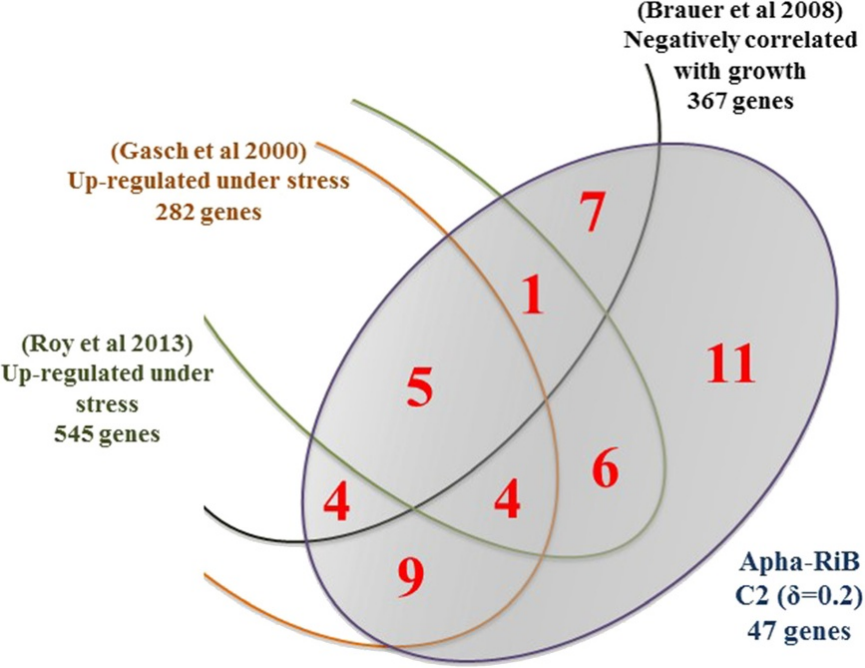
**Venn diagram showing the size of overlap between our novel APha-RiB cluster (C2 at DTB with δ = 0.2) and the subsets of genes with expression reported to be positively correlated with stress and negatively correlated with growth in three previous studies** [22, 23, 60] **.**

The phenomenon of opposite co-expression of RRB and stress response genes in budding yeast was reported by various studies [22, 23, 59, 60]. As shown in Figure 9, the subsets of genes identified by the studies of Gasch (2000) [60], Brauer (2008) [22], and Roy (2013) [23], and their collaborators are much larger than the APha-RiB regulon defined in our study (hundreds of genes versus 47 genes). Moreover, the largest overlap between any of those subsets of genes and APha-RiB does not reach half of the genes in APha-RiB, where the largest overlap, which is between APha-RiB and the subset identified by Gasch and colleagues [60], includes 22 genes. Furthermore, none of those previously reported, relatively large, subsets includes more than two of the eight genes highlighted for their importance in Figure 7 and Figure 8, and discussed below. This illustrates the novelty of this focused and specific cluster which has been found by our large scale genome-wide analysis of forty different and recent datasets.

Taken together, firstly, we have observed and reconfirmed the reciprocal behaviour of RRB and some genes participating in stress response over datasets which cover much wider conditions including ones that are not directly related to stress changes, e.g. cell-cycle datasets. Secondly, our APha-RiB subset of genes consistently reciprocally expressed with RRB largely includes genes with unknown or apparently unrelated biological processes, in addition to few genes known to participate in stress response. Thirdly, our method does not require that the microarray samples are combined into a single dataset, in contrast to the studies by Gasch [60] and Brauer [22] and their colleagues. It is therefore now possible to analyse large number of datasets in the literature in a single experiment, even if the datasets are diverse in time, location, condition, and use different microarray platforms. Finally, although a proportion of the APha-RiB genes has been explicitly associated with response to oxidative stress processes (six out of 47 genes), the processes in which the rest of the genes in APha-RiB participate are either unknown or apparently unrelated. Additionally, the forty datasets considered in this study cover a much wider range of stress and growth conditions than oxidative stress. Given that, most of the genes in APha-RiB are yet to be associated with biological processes and/or their function to be understood within the context of generic, not specific, stress response; our results suggest these areas would be the subject for fertile future investigation.

### Proposed model for the transcriptional regulation of RRB and APha-RiB

**Figure 10:**
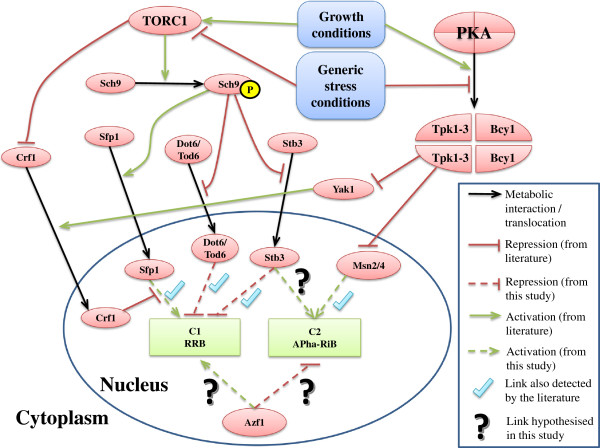
**Regulation of the RRB cluster (C1) and the APha-RiB cluster (C2).** Ticked dashed links have been detected in this study and were also previously identified in the literature while dashed links with question marks have been only detected in this study. However, most of the previous studies consider one or few stress conditions in contrast to “generic stress conditions”. Notice that the cluster “C2 APha-RiB” is novel and that the links from the literature that point at it are based on the assumption that it is a stress response module.

The temporal expression of the cluster APha-RiB (C2) in opposite direction of regulation to the RRB genes (C1), as well as the high enrichment of common motifs in the upstream DNA sequences of genes in APha-RiB (Figure 5), strongly support the hypothesis that genes in the subsets RRB and APha-RiB are regulated by the same biological machinery, or possibly that the transcriptional regulators for both clusters are regulated by a common regulator. Therefore, we propose an outline model of regulation for the genes included in RRB and APha-RiB clusters (Figure 10).

The model in Figure 10 shows parts of the TOR and the PKA signalling pathways which are regulated by the presence of some growth factors (e.g. glucose) or the presence of some stress conditions, and then they regulate RRB and stress response modules of genes. Although we use the general terms “growth conditions” and “generic stress conditions” instead of more specific terms such as “glucose abundance”, “oxidative stress”, most of the previously discovered links of regulation were in the context of one or few growth conditions such as the presence of glucose [14, 61, 62], ammonium [14], or other specific nutrients, or to types of stress such as oxidative stress [63] or methyl methanesulfonate (MMS) DNA-damage stress [64]. However, using such general terms here reflects the comprehensive nature of the data analysed by the Bi-CoPaM approach as we have been able to consider and analyse a wide range of different growth and stress conditions in a comprehensive and systematic way. Indeed, we can now reach a consensus conclusion, that up- and down-regulation of the RRB and APha-RiB clusters are influenced by a wide range of growth and stress conditions (Table 1).

Many of the direct regulators detected in this study by upstream sequence analysis of the RRB and the APha-RiB subsets of genes (dashed links in Figure 10) were also previously identified in the literature (ticked dashed links). Indeed, the regulatory links from the literature to the novel APha-RiB cluster are based on the assumption that it is a stress response subset of genes.

It could be argued that one of the two clusters actually negatively regulates the other. This seems unlikely for several reasons. First, the synchronisation between both clusters is very high such that there is insufficient phase shift between them for one to regulate the other. Second, the functionality of a transcription factor is likely to be regulated post-translationally in many ways, such as the existence of another metabolite or signal, localisation changes, or others [62, 65]. It is doubtful that many regulators could be functionally active in a consistently similar profile for a very large number of target genes. Therefore, we would suggest that these two clusters of genes are transcriptionally regulated by common machinery rather than one of the clusters transcriptionally regulates the other.

It could also be hypothesised that the two clusters are regulated by two separate pathways that are oppositely activated in synchrony with growth and stress conditions. Though, this hypothesis necessitates that those two transcriptional regulation pathways are consistently and synchronically regulated by various types of growth and stress signals, or that those signals regulate a single signalling pathway which regulates both transcriptional regulatory machineries. In this case, the common upstream regulator of the two clusters would be a signalling pathway or the signals themselves. Although this is a possible proposal, the fact that the signals that consistently and synchronically regulate both groups are largely variant, we focus on the hypothesis that both groups are regulated by a common machinery, or that their regulatory machineries regulated by a common regulator. Indeed, the latter proposal conforms to the more general statement of Brauer and colleagues that such consistent positive or negative correlation reflects system-level regulatory mechanisms [22].

### Potential regulators for APha-RiB and common regulators for RRB and APha-RiB

Gasch and Roy and their collaborators commonly identified the Msn2p and its paralogue Msn4p as regulators for the subsets of genes which they identified as negatively correlated with growth [23, 60]. Gasch and colleagues also identified Yap1p as a regulator for their group [60] while Roy and colleagues identified Rtg1p and Adr1p [23]. Interestingly, upstream analysis for our novel cluster APha-RiB (C2) has identified Azf1p and the paralogous pair Msn2p and Msn4p as potential regulators (Figure 5). It is worth noticing that the three studies mutually identify Msn2p and Msn4p, which are well known for their role in stress response regulation through binding to the STRE motif (Figure 10) [66, 67].

More interestingly, Azf1p has been identified by our results as a potential regulator for in both clusters RRB (C1) and APha-RiB (C2) (Figures 4, 5, and 10). Azf1p is a zinc-finger transcription factor, which has been predicted to have role in one of the putative stress response regulatory modules [68, 69]. Moreover, it is exclusively localised in the nucleus and it was found to be synthesised in higher amounts under non-fermentable growth conditions [70]. By monitoring differentially expressed genes when AZF1 was knocked down, Slattery and colleagues showed that this gene’s product participates in the transcription of two non-overlapping subsets of genes under two different conditions. The common aspect between these non-overlapping subsets of genes is having the motif AAAAGAAA in their promoters [71]. Although our C2 genes at δ = 0.2 are not included in any of these two subsets, the existence of the AZF1 binding site in their promoters indicates that AZF1 may regulate expression of genes in this cluster under other conditions.

Another candidate common regulator is Stb3p (Figure 10), which binds to the consensus motif TGAAAAA [61, 62, 72]. This motif largely overlaps with the RRPE motif found in the upstream sequences of the RRB genes in our results, as identified by the TOMTOM tool (Figure 4(B)). Although not identified by the TOMTOM tool as a potential binding transcription factor, its binding motif TGAAAA largely overlaps with the part of the motif C2-1 (Figure 5). Moreover, Stb3p overexpression was shown to increase resistance to oxidative stress [63] and to result in down-regulation of ribosome biogenesis genes [61, 62, 72], and Liko and colleagues also predicted that Stb3p would be expected to regulate transcription of other unknown sets of genes positively [61, 62].

The evidence for Azf1p or Stb3p acting as a transcription activator and/or repressor with relation to both groups of genes – RRB genes (C1), and APha-RiB genes (C2) is unclear. Nevertheless, there are enough observations to speculate that one of them or both of them may play a role in the mutual transcriptional regulation of both RRB and APha-RiB. The molecular mechanism(s) and significance of those transcription factors in this context remain to be established.

### A subset of eight genes in the APha-RiB cluster are highly connected across various gene networks

Strikingly, a novel subset of eight out of the 47 genes in APha-RiB (C2 at DTB with δ = 0.2) have shown high connectivity in co-expression, protein-protein physical interactions, and genetic interactions (highlighted in Figure 7 and Figure 8). The genes YIR016W, AIM19, and OM14 have unknown biological processes. The latter two localise in the mitochondria while the localisation of YIR016W is unknown. UGA2 is an oxidative stress response gene which localises in the cytoplasm. PMP3 is a plasma membrane gene that participates in response to drugs and regulation of membrane potential. YOR228C’s product is a mitochondrial protein which is involved in lipid homeostasis but with an unknown function. NCR1’s product is a vacuolar membrane protein which participates in vacuolar protein sorting pathway. Finally, YSA1’s product participates in ribose phosphate metabolism and was found localising in the mitochondrion, cytoplasm, and the nucleus. Clearly, those genes, generally, have unknown or apparently unrelated functions despite this high connectivity.

One focal gene with previously unknown function is YIR016W. Large scale overexpression screening in yeast revealed that this gene’s overexpression causes cell-cycle to be arrested by accumulating cells at the G2/M stage [73], which is consistent with its down-regulation during the cell-cycle as shown in our results (e.g. datasets D01, D02, D21, and D22 in Figure 3). Arresting the cell-cycle under stress is one of the known mechanisms for stress response [9]. Its co-expression, protein-protein physical interaction, and negative genetic interaction with the stress response gene UGA2 strengthens the hypothesis that this gene may participate in stress response, and its connections with UGA2, YSA1, and AIM19 provide a concise platform for future functional studies. This gene’s role in cell-cycle arrest/delay and in any other mechanisms of stress response has to be revealed in future functional studies.

The highly consistent up-regulation under stress, down-regulation under growth, and high network connectivity for this novel and concise subset of genes across such wide range of conditions in forty different datasets indeed indicate that they have roles in some of the mechanisms related to generic stress response. After scrutinising this sub-network, as well as the APha-RiB cluster in general, it becomes clear that many details regarding generic stress response mechanisms and their member genes are yet to be elucidated.

### The Bi-CoPaM method is useful for genome-wide consistently co-expressed genes discovery

Our results have demonstrated the usefulness of our novel approach of using the Bi-CoPaM method to explore genome-wide expression data, from various microarray datasets from different biological contexts and conditions. More specifically, we have defined subsets of genes that are consistently co-expressed across various microarray datasets using a tunable method and without the need for *a priori* knowledge-based filtering.

In contrast to other clustering and ensemble clustering methods, configuring the Bi-CoPaM method to generate sixteen clusters does not imply that the final objective is to get sixteen informative clusters; the final objective is rather to mine for the few subsets of genes which are consistently co-expressed in all or most of the considered datasets [18]. A larger number of clusters than expected to be informative is required to account for the large variation in the genome-wide expression. In Bi-CoPaM, the genes that are consistently co-expressed in all or most of the datasets when considered by various clustering methods will constantly appear in the same cluster. The majority of genes, which will show low consistency in co-expression, are allowed a wide space of sixteen clusters to be assigned to. These genes will appear in different clusters when their expression profiles indifferent datasets are considered from by the various clustering methods. Therefore, inconsistency in co-expression is reflected by inconsistency in cluster assignment.

Our results have demonstrated that a wide range of numbers of clusters (K) will result in the same tight clusters. The Bi-CoPaM’s difference threshold binarisation (DTB) technique tunably tightens the clusters to include the most consistently co-expressed genes while leaving the large bulk of the poorly co-expressed genes unassigned from all of the clusters [19, 20]. This tunable tightening is controlled by the parameter δ, which increases the tightness as it is increased. As can be seen in Table 2, most of the sixteen clusters lose all of their genes at relatively low δ value, which is to be expected as most of the genes will not be co-expressed in most of the studies and datasets considered.

In their 2001 study, Wade and colleagues clustered about half of the genome into 24 clusters from three different datasets by a single clustering method, which is the partitioning around medoids (PAM) method [74]. They then performed statistical analysis to identify overlapping clusters from different results, which led to finding that only one cluster from one dataset has significant overlap to another cluster from one other dataset. The intersection between both clusters had 65 genes, which were found to be largely participating in ribosome biogenesis [74].

When comparing the two approaches, three major differences are the most important. First, our approach is more suitable when larger numbers of datasets are considered because of the systematic way of fusing the results into a single consensus result that reflects all information. Second, Bi-CoPaM allows for various crisp and/or fuzzy clustering methods to be applied over each single dataset, which adds another level of diversity. Third, and most importantly, our Bi-CoPaM-based approach is tunable and is not merely limited to the intersections of clusters; if direct intersection worked well for two datasets in Wade’s approach, it would result in empty clusters in the case of forty datasets with various clustering methods. This can be directly and clearly seen in our results as intersection is a special case of Bi-CoPaM’s results, and is obtained by DTB with δ = 1.0, the case at which all of the sixteen clusters have been found completely empty (Table 2). On the other hand, considering conventional complementary clusters, which is again a special case of the Bi-CoPaM results (DTB with δ = 0.0), is impractical as it does not reduce the complexity of the datasets, and the clusters at this level are generally looser than acceptable (see Figure 2 and Table 2). Therefore, the most fruitful analysis, as demonstrated by our study, is when clusters are tightened while maintaining significant numbers of genes and here the Bi-CoPaM approach allows observation of clusters’ behaviour when δ parameter is tuned to produce tight clusters in Table 2 and Figure 2.

Taken together, our approach can analyse large amounts of high-throughput datasets to produce relatively focused and comprehensible results that capture the most consistent aspects of the raw data. The method can therefore discover those subsets of genes most consistently co-expressed under various conditions.

### Conclusions

We have applied the Bi-CoPaM method over genome-wide data from forty microarray datasets with wide range of different biological contexts and experimental conditions in order to identify the subsets of budding yeast genes that are most consistently co-expressed. We found two clusters of genes that have significant consistency of co-expressions, which we have labelled as RRB (C1) and APha-RiB (C2). These two clusters preserved their status as the tightest two clusters at varying values of K, which shows their importance as well as the robustness of the proposed Bi-CoPaM approach. By GO analysis, C1 has been found to be highly enriched with ribosome biogenesis and rRNA processing (RRB) genes. On the other hand, most of the genes included in C2 have unknown or apparently unrelated functions.

Finding RRB genes (C1) in the tightest cluster by this completely unsupervised approach, confirms not only that these genes are consistently co-expressed under various conditions [9], but also that they are the most consistently co-expressed genes across the whole genome. Additionally, our C1 cluster includes few genes with unknown processes that may be worthy of biological investigation.

The most interesting cluster of genes in our results appears to be C2, and this is for three main reasons – first, these genes are mostly unknown or apparently unrelated to each other, despite the fact that they are the second most consistently co-expressed subset of genes in budding yeast; second, their average expression profiles show consistently anti-phase (opposite) expression to the average expression profiles of RRB genes (C1) across all of the forty datasets; and third, significant genetic and protein-protein physical interactions have been reported between them by high-throughput studies in the literature. These observations lead us to label C2 as the subset of genes in *anti-phase with ribosome biogenesis (APha-RiB)*, to suggest that many of the unknown genes in APha-RiB (C2), such as YIR016W, may participate in different generic, in contrast to specific, stress response mechanisms, and to suggest that RRB genes (C1) and the APha-RiB genes (C2) may be transcriptionally regulated by common machinery or that their regulation machineries may be controlled by common post-translational regulators. We have identified potential factors that might be involved in such reciprocal regulation, for example Azf1p and Stb3p.

This study has yielded globally consistent co-expression in budding yeast and produced new, focused insights for future work to elucidate and confirm the components of the common regulatory machinery for RRB and APha-RiB, and to define the function of poorly characterised genes in both clusters. The results from the application of the Bi-CoPaM method to yeast datasets strongly suggests that it may be helpful for the analysis of other groups of microarray datasets from other species and systems for the exploration of global genetic co-expression.

## Electronic supplementary material

Additional file 1: Table S1: A list of the 5667 genes included in this study and the contents of all of the sixteen clusters at all of the adopted δ values. (XLSX 261 KB)

Additional file 2: Figure S1: Provides the profiles of the genes included in the clusters C1 and C2 at the tightness levels of DTB with δ = 0.3 and 0.2 respectively. The profiles are provided from all of the forty considered datasets. (PDF 1 MB)

Additional file 3: Table S2: GO Term analysis results of the processes enriched in the clusters C1 and C2. (XLSX 136 KB)

Additional file 4: Table S3: GO Slim analysis results of the processes enriched in the clusters C1 and C2. (XLSX 69 KB)

Additional file 5: Table S4: GO Term and GO Slim analysis results of the cellular components enriched in the cluster C2 at δ = 0.2. (XLSX 15 KB)

Additional file 6: Figure S2: C1 overlap with similar clusters in the literature, and the ratios of included genes associated with the “ribosome biogenesis” GO term. (PDF 354 KB)
