# Supplementary material for: Comprehensive analysis of forty yeast microarray datasets reveals a novel subset of genes (APha-RiB) consistently negatively associated with ribosome biogenesis
Source: BMC Bioinformatics. 2014 Sep 29;15(1):322. doi: 10.1186/1471-2105-15-322 (PMC4262117; doi:10.1186/1471-2105-15-322)
Supplement: Supplementary file 2 — Additional file 2: Figure S1: Provides the profiles of the genes included in the clusters C1 and C2 at the tightness levels of DTB with δ = 0.3 and 0.2 respectively. The profiles are provided from all of the forty considered datasets. (PDF 1 MB) [file 12859_2014_6633_MOESM2_ESM.pdf]

Supplementary Figure 1

This Supplementary Figure provides the profiles of the genes included in the clusters C1 and C2 at the tightness levels of DTB with δ = 0.3 and 0.2 respectively. The profiles below are provided from all of the forty considered datasets.

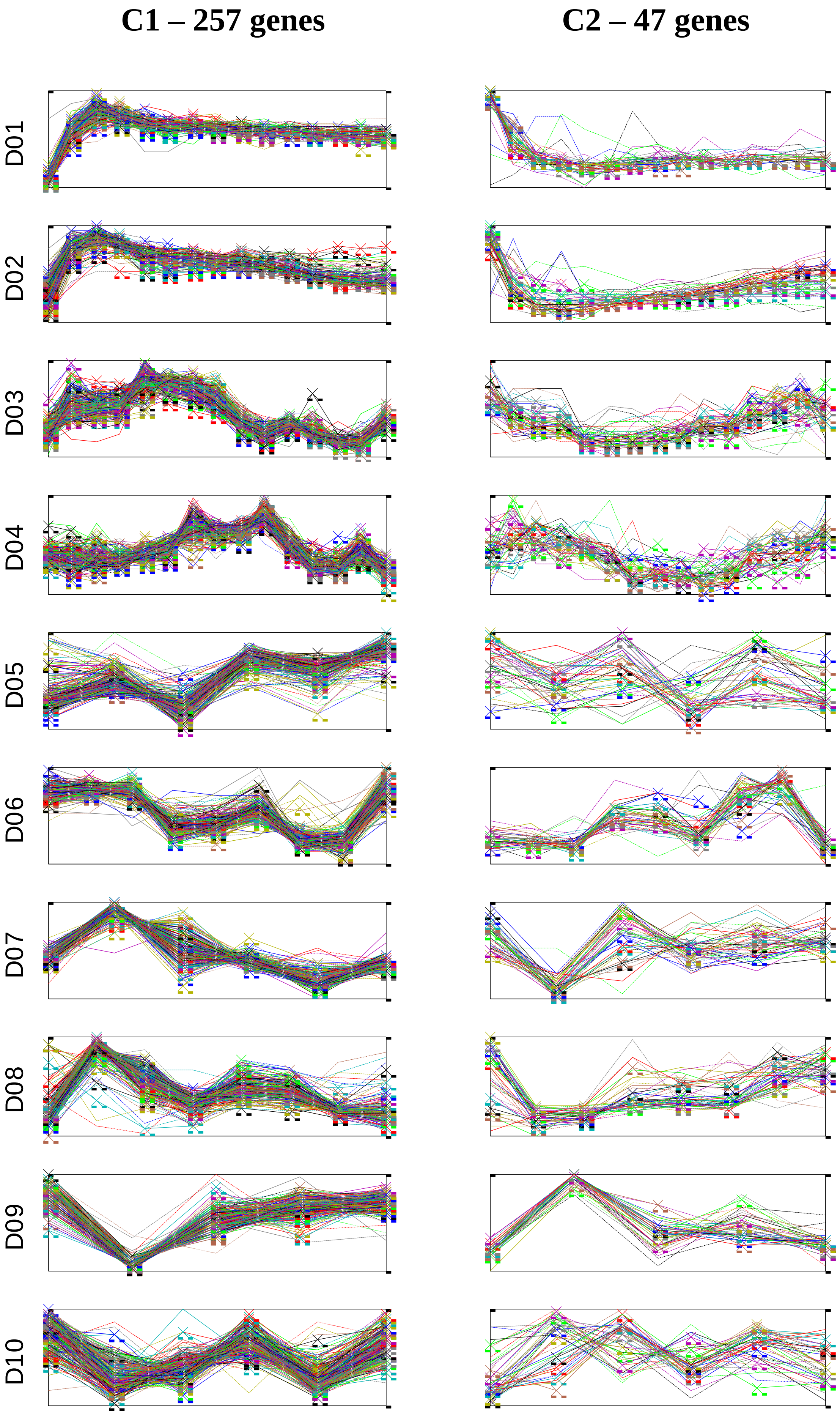

C1 – 257 genes
C2 – 47 genes
D01
D02
D03
D04
D05
D06
D07
D08
D09
D10

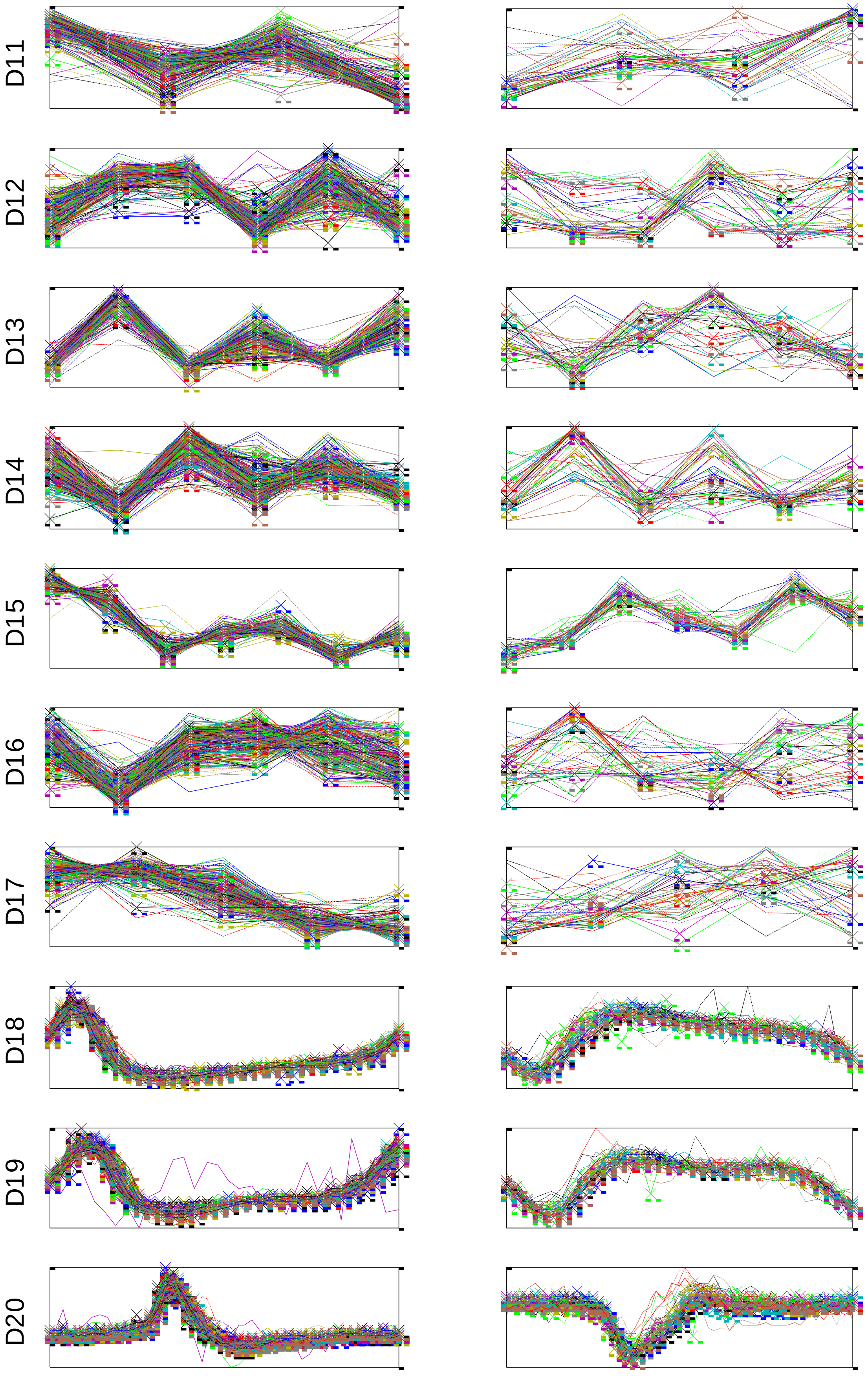
D11
D12
D13
D14
D15
D16
D17
D18
D19
D20

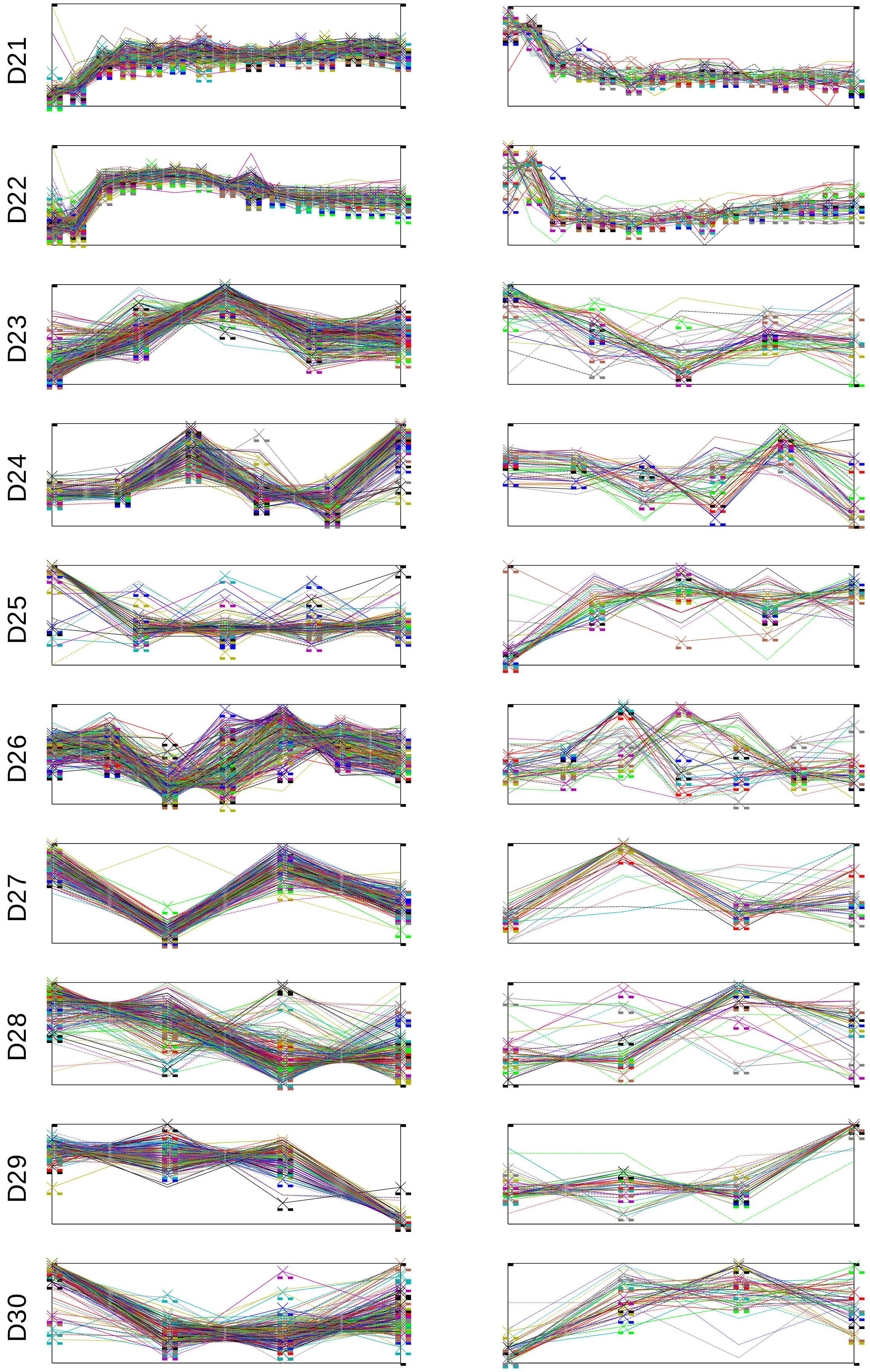
D21
D22
D23
D24
D25
D26
D27
D28
D29
D30

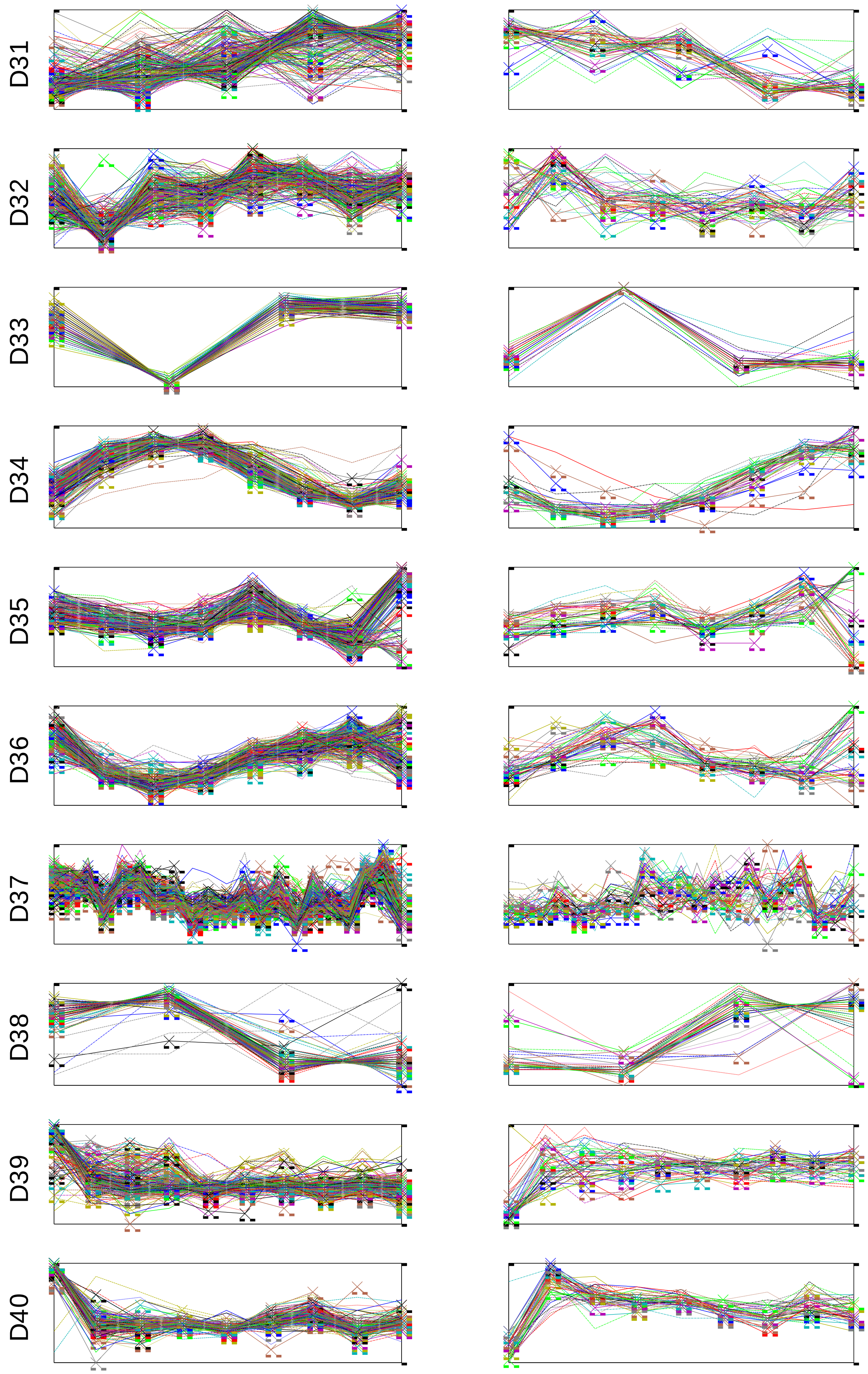
D31
D32
D33
D34
D35
D36
D37
D38
D39
D40
