# Supplementary material for: Comprehensive analysis of forty yeast microarray datasets reveals a novel subset of genes (APha-RiB) consistently negatively associated with ribosome biogenesis
Source: BMC Bioinformatics. 2014 Sep 29;15(1):322. doi: 10.1186/1471-2105-15-322 (PMC4262117; doi:10.1186/1471-2105-15-322)

Supplementary Figure 2

C1 overlap with similar clusters in the literature, and the ratios of included genes associated with the “ribosome biogenesis” GO term.

(Wade et al. 2006) – RRB regulon

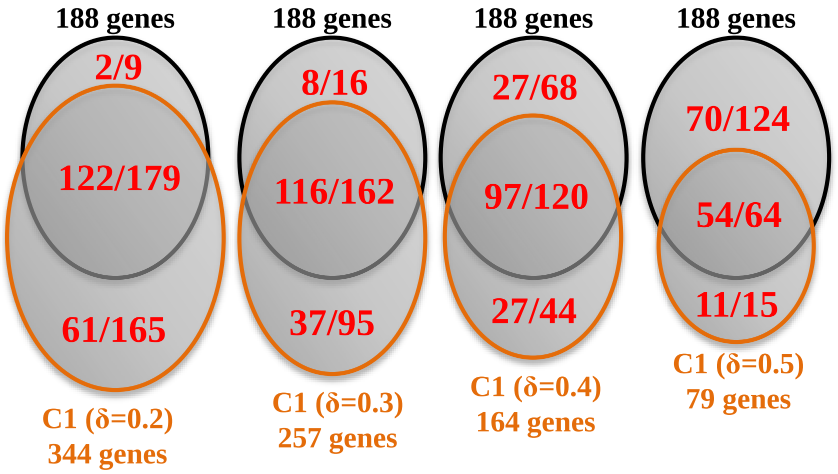

(Brauer et al. 2008) – Positively correlated genes with growth

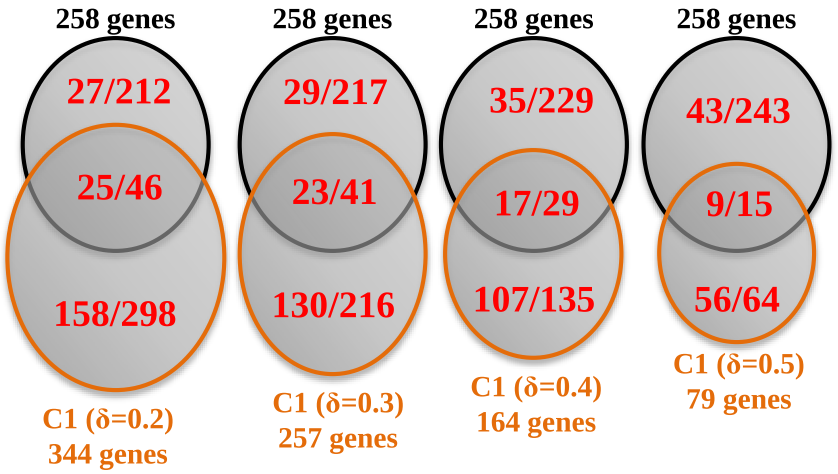

(Roy et al. 2013) – Down-regulated genes with stress

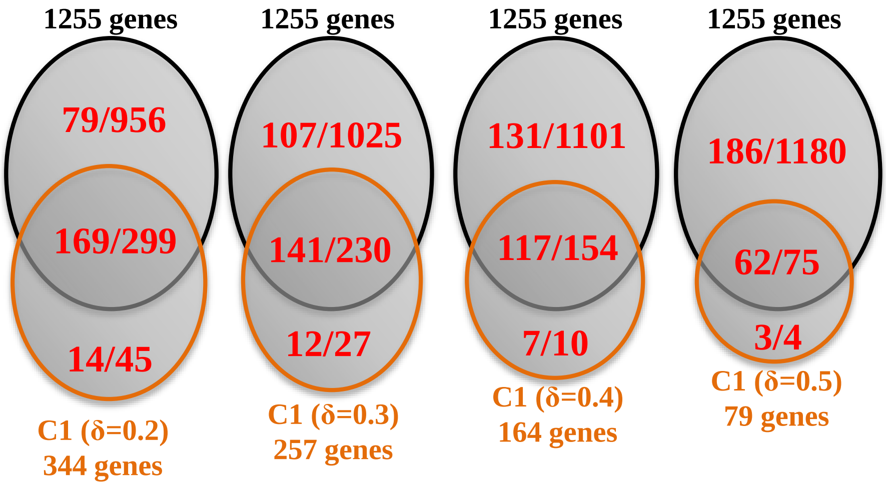

Supplement: Supplementary file 6 — Additional file 6: Figure S2: C1 overlap with similar clusters in the literature, and the ratios of included genes associated with the “ribosome biogenesis” GO term. (PDF 354 KB) [file 12859_2014_6633_MOESM6_ESM.pdf]
